# Medical and Philosophical Intelligence

**Published:** 1817-04

**Authors:** 


### MEDICAL AND PHILOSOPHICAL INTELLIGENCE.

[The following short papers we have found most conveniently disposed of in this department of our Journal.]

*For the London Medical and Physical Journal.*

*Queries regarding Dr. Ley's Case of Puerperal Fever;* by MEDICUS.

THE unusual and alarming occurrence of a case of Puerperal Fever proving fatal after an easy natural labour, the woman being in good health at the commencement of her labour, and the disease not being prevalent in the house, (the Westminster Lying-in Hospital, where the case occurred,) renders it (to me at least, and, perhaps, others of your readers may view it in a similar light) a question of some importance, practically considered, to ascertain, whether it be an established rule of the Hospital to administer an opiate after every labour, whether difficult or otherwise; and, if not, not, whether some symptom, not mentioned in the narrative, occasioned an opiate to be administered in the case alluded to?

"Nothing untoward," it is said, "occurred in her delivery. Her labour was, in every respect, perfectly natural, the dilatation of the os uteri being rapidly progressive, the presentation most favourable, and the external parts easily dilatable. As soon as the division of the funis had been effected, and the infant delivered to the ordinary attendant, it was ascertained that the placenta had been separated by the contractions of the uterus, and was already lodged in the vagina. In a few minutes it was wholly expelled by the natural efforts of the woman, no assistance having been given by the funis. An opiate having been administered, she was put to bed." &c.*

I am aware, that, as the case was not originally published in your Journal, (being taken from the Medical Transactions,) it is at the option of the ingenious author of the narrative either to give or withhold the information desired: but, as mere curiosity has no share in inducing me to make the inquiry,—my motive being of a general nature, and with a view to utility,—I flatter myself my request will be attended to.

*Manchester; Feb.* 21, 1817.

---

*To the Editors of the London Medical and Physical Journal.*

GENTLEMEN,

On reading the well-merited eulogium on Dr. Rush, contained in your Journal for January, I could not avoid contrasting the opinion entertained of his sagacity and discernment by a Lettsom, and a Zimmerman, with the affirmation of a late writer: "it is evident that, in the year 1794, he mistook the Bilious Remittent for the Bulam Fever."

This singular assumption, to get over a difficulty, is about as well-founded and legitimate a conclusion as that "the Sydenham of America," in disavowing contagion, and a living physician of pre-eminent experience, are entitled to *less* credit, because they have had the candour to acknowledge that they had been in error, and had changed their opinion;—or as the "discovery" that the yellow-fever attacks but once; an hypothesis (like the Bulam or error loci theory) the adoption of which it might have been expected that a very moderate *personal* acquaintance with the disease, in different situations, would have prevented. The "fact" seems lately to have been correctly stated by Mr. Doughty and others, to wit, that the liability to a second attack will depend upon the superior intensity of cause, or regenerated susceptibility, by intervening ultra-tropical residence. P. 182-3.—I am, &c.

*London; Feb.* 12. ÆQUUS.

The writer of the above wishes to ask those who have read the

---

* Med. and Phys. Journal, vol. 37, p. 117, &c.

French

French calumny, whether they have seen the recantation of Dr. Rush? because it is presumed that no one could peruse the language of solemn and deep contrition in which it is couched, and doubt his sincerity for a moment,—under any thing short of absolute proof,—without being deficient in one of those hitherto conceived necessary appendages, a head or a heart.

---

*To the Editors of the London Medical and Physical Journal.*

GENTLEMEN,

WITH much surprise I have seen it stated, in Mr. Pettigrew's Memoirs of the Life and Writings of the late Dr. Lettsom, that "the vaccine lymph was first sent by him across the Atlantic, and consigned to the fostering care of Dr. Waterhouse, Prof. of the Theory and Practice of Medicine in the University of Cambridge, Massachusetts; and that from him it was spread through the United States." But this is not true; vaccine lymph had been previously sent by Dr. George Pearson to Dr. John Chichester, now a resident physician at Bath, but at that time in very extensive practice at Charleston in South Carolina. An opportunity soon offered for the employment of it, of which Dr. C. availed himself, and the particulars may be seen by reference to Mr. Tilloch's Philosophical Magazine, vol. 16, page 252, where they are stated by Dr. Rhodes, who witnessed the insertion of the matter, and the progress of the vaccine disease throughout its whole course. The subject was afterwards tested with variolous matter, and the constitution found proof against its effects. Your's,

*London; March* 13, 1817. GEORGIENSIS.

---

*To the Editors of the London Medical and Physical Journal.*

GENTLEMEN,

THE oil of bitumen, or coal tar, is considered by those who make and burn gas as waste; but mix coal tar with dry saw-dust, spent logwood, or fustic, to the consistence of paste, and let the same remain until the water is drained off; and, if 2 cwt. of the mass be put into the retort instead of coals, it will produce more gas, and be less offensive, than the same weight of canal coal. It may be thus repeated until the whole of the tar is consumed into gas. This will not only be a saving of about one half the expence of coals, but will add to cleanliness and neatness. Consuming the residuum of hydrogen gas on the above plan cannot be too strongly recommended, as it is well known to have a very offensive odour.

ARCHIBALD BEECH, *Chemist.*

23, *London Road, Manchester;*
*May* 21, 1816.

*To the Editors of the London Medical and Physical Journal.*

GENTLEMEN, *Feb.* 27, 1817.

WHEN I first sent you my Calendar of Flora last year, I had occasion to remark the great backwardness of the spring; and, I believe, from the appearance of the present season, that we may expect it will afford us an opportunity of contemplating an equally early produce. This has induced me to summons together my botanical friends, in order to collect the flowers that are now open. Our excursion this day was through Battersea Fields; and the following is a list of what we procured.

| | |
|---|---|
| Leontodon Taraxacum | Primula veris |
| Alsine media | Poa annua |
| Veronica agrestis | Helleborus fœtidus |
| Cerastium vulgatum | —— viridis |
| Lamium purpureum | Caltha palustris |
| Daphne Laureola | Tussilago Petasites |
| —— Mezereum | Viola odorata |
| Crocus vernus | Anagallis arvensis |
| —— nudicaulis | Thlaspi Bursa Pastoris |
| Galanthus nivalis | Senecio vulgaris |
| Cheiranthus fruticosus | Saxifraga oppositifolia |
| Taxus baccata | Ranunculus Ficaria |
| Buxus sempervirens | Lamium amplexicaule |
| Euphorbia Helioscopia | Veronica hederifolia |
| Viola tricolor | Lamium album |
| Bellis perennis | Tussilago Farfara |
| Corylus Avellana | Draba Aizoides. |

*March* 19th.—This day I made another excursion to the same place, and we found the following plants, in addition to the above.

| | |
|---|---|
| Salix viminalis | Ulex europæus |
| Glechoma hederacea | Ribes rubrum |
| Equisetum hyemale | —— Grossularia |
| Draba verna | Urtica urens |
| Cerastium viscosum | Narcissus Pseudo Narcissus. |

It will be curious to contrast the time of opening of all these early flowers with that of the last spring; and, should the weather continue as fine as it appears to be at present, my regular list (which I hope to give you every month) will afford you some interesting facts, from whence, I doubt not, many may reap amusement by contrasting the difference of the two seasons.

I am, Gentlemen,
Your obedient Servant,
WILLIAM SALISBURY.

I shall make another excursion on the second Thursday in April, to Hampstead Heath; and on the third Thursday to Battersea Fields,—to meet at ten in the morning.

List

***List of Patients admitted into all the Civil Hospitals of Paris, from the* 1*st of February, to the* 1*st of March*, 1817.**

| | |
|---|---|
| Uncharacterised fevers | 15 |
| Intermittent ditto of various kinds | 142 |
| Bilious or gastric ditto | 85 |
| Mucous ditto | 7 |
| Adynamic or Putrid ditto | 20 |
| Ataxique | 1 |
| Catarrhal ditto | 7 |
| Phlegmasies, internal or external | 113 |
| ——— of the organs of respiration | 57 |
| Consumptions | 24 |
| Ophthalmia | 27 |
| Diarrhœa and dysentery | 17 |
| Dropsy and anasarca | 13 |
| Apoplexy and recent paralysis | 16 |
| Colliques metalliques | 4 |
| Small-pox | 1 |
| Sporadic and chronic disorders, and the result of accidents | 284 |
| Itch | 74 |
| Total | 827 |

In the above, the number of intermittents is truly surprising, and also the absence of scarlatina and measles.

*Of Blows upon the Nails.*—One of the most painful of minor accidents is a blow upon the nail. The immediate effect is to produce a violent pain, which, instead of diminishing, increases for several hours. This pain is produced from the blood which has escaped from the little bruised vessels collecting under the nail, and forms those black spots which are almost always observed in these cases. As the blood thus collected has no means of escaping, an inflammation follows at the end of the finger, and afterwards the formation of a purulent matter, and at length the nail falls off in the midst of excessive pain; frequently the new nail produced is deformed, because the disorder has altered the structure of the parts which reproduce it.

There is, however, a method of preventing all these accidents, or at least greatly diminishing them; this consists only in giving vent to the blood by piercing the nail; this little operation is perfectly similar to trepanning. It is useless to point out to medical men the mode of operation, but, to others, the following easy method is within the reach of every one:—Scrape the nail on the black part with a piece of glass till you arrive at the pellicle, which must be pierced with a pen-knife. At the instant black blood will rush out, and the pain will be relieved; all the dressing necessary is a linen bandage to receive the blood, which will continue to exude, and Nature perfects the cure.

If the blow has been violent, and the nail entirely loosened, it will fall off without causing much pain; but it may happen, that, notwithstanding the evacuation of the blood by the incision of the nail, the finger will inflame, it will then be necessary to bind up the finger with a bread and milk or water poultice, which will dissipate the pain and the inflammation.

---

### *On the Mystic Forms and Studied Obscurity of the Formulæ of the Ancient Physicians;* by Dr. Montegre.

The number of physicians having greatly increased at Rome, several of them endeavoured to captivate the attention of the multitude, who, according to Pliny, believe the more as they comprehend less, (minus credunt quæ ad suam salutem pertinent, si intelligunt.) Some published their prescriptions in an enigmatic style, either to conceal their knowledge from the vulgar, or to impose on them the more. We may quote, for example, the Philonium, of which the author describes the composition in Greek verses, which have been preserved by Galen, (De Composit. Medicam. Local, l. ix. de Sedantibus.) "Take (says Philo) as many drams as man has senses of the red and odorous hairs of the child whose blood is yet spread in the fields of mercury, (crows killed by mercury in playing at the Discus;) a drachm of Euboci Nauplium, (the plant of the fire designated under the name of Nauplius, who lighted large fires on the coasts of Eubœa to deceive the Greeks on their return from Troy;) a drachm of the murderer of the son of Menoctius, (Euphorbus, who first wounded Patroclus;) add twenty grains of white flame (white pepper;) an equal weight of the beans of the hogs of Arcadia, (hyoscyamus signifies in Greek, hog-beans; the boar of Erymanthea, killed by Hercules, had been fed in Arcadia;) an equal quantity of the plant, falsely called root, brought from the country renowned on account of Jupiter Pissean, (spikenard came from the Isle of Crete;) write pium, and add to the head of this word the masculine article of the Greeks *(o)*; take twice five drachms, and mix the whole carefully with the work of the daughters of the Bull of Athens (bees)." Thus, in plain terms, this mystic recipe is simply to take certain quantities of saffron, the root of the pyrethrum, (pellitory of Spain,) euphorbium, white pepper, henbane, spikenard, and opium, and mix the whole with attic honey. This remedy, according to the author, cured the colic, pains of the liver, pains of the stone and bladder, disorders of the spleen, difficulty of breathing, convulsions, ethisia, pleurisy, spitting and vomiting of blood, all internal pains, coughs, hiccough, &c. &c. &c. It may easily be conceived, that, to inspire the belief of the efficacy of this remedy in all these disorders, it was necessary not to make it too public.

---

*Rheumatism and Rheumatic Gout.*—Light infusions of ginger alone, taken twice or thrice a day, has been found very efficacious by the French surgeons in rheumatic affections. The pains are rendered at first more excruciating; then follows copious perspiration; ration; and, in two or three days, the symptoms gradually disappear, and the patient is cured, or at least relieved for a period.

---

*Necessity of Food containing Azote for the Nourishment of Animals.*—It is a commonly received opinion that sugar, gum, oil, butter, and some other similar bodies, which contain no azote, constitute very nourishing articles of food. This opinion has been put to the test of experiment by M. MAJENDIE. A dog was fed upon sugar and distilled water. He eat his food readily, and for some time retained his health and usual liveliness. But in about a fortnight became lean, though his appetite continued good. His alvine excretions were scanty, but his urine was abundant. On the twenty-first day an ulcer began to appear in the centre of the cornea of each eye, which gradually increased, penetrated the cornea, and the humours of the eye ran out. The leanness continually increased, the animal lost its strength, and died on the thirty-second day. The dead body was found destitute of fat, and the muscles deprived of five-sixths of their usual volume. The urine was alkaline, and destitute of uric acid, like that of herbivorous animals. The bile also contained much picromel, like that of oxen. A second and a third dog, fed likewise upon sugar and water, shared a similar fate.

Two dogs fed upon olive oil and water died on the thirty-sixth day, with precisely the same phenomena, except the ulceration in the cornea, which did not take place.

Several dogs were fed with gum and water. Their fate was precisely the same.

A dog fed on butter died on the thirty-sixth day, with precisely the same phenomena; though, on the thirty-fourth, flesh was given him, and he was allowed to eat of it at pleasure. In him, the ulceration in the cornea showed itself.

From these curious experiments of M. Magendie, it is obvious that none of these articles are capable of nourishing dogs. Hence we may infer, with the greatest probability, that they are incapable of nourishing man. (Ann. de Chim. et Phys. iii. 66.)

---

*Discovery of the Existence of Cobalt in Meteoric Iron.*—The existence of nickel and chromium in meteoric stones has long been known; and an experiment of Klaproth (Beitrage, vi. 297) leads to the suspicion of the existence of cobalt in the same minerals. The existence of cobalt in the meteoric iron from the Cape of Good Hope has been ascertained by Professor Stromeyer, who lately analysed a specimen of it sent him by Mr. Sowerby. He was unable to detect cobalt in the meteoric iron from Siberia and Bohemia; but he considers the methods at present used to separate cobalt from nickel as bad, and he has not yet discovered a better.

---

*Death of Klaproth.*—On January 1, 1817, MARTIN HENRY VON KLAPROTH, Professor of Chemistry at Berlin, and by far the most most celebrated chemist in Germany, died at a very advanced age. He has been a distinguished writer for at least forty years; for he published a set of chemical experiments on Copal in the year 1776. Chemistry lies under greater obligations to him than to any other chemist of his time. He devoted himself entirely to analytical chemistry; and to him we are chiefly indebted for the knowledge which we at present possess of the mineral kingdom, and for the formulas employed to develope the constituents of minerals. His labours are consigned in six octavo volumes, under the title of Beitrage zur chemischen Kenntniss der Mineralkorper, the first volume of which was published in 1795, and the last in 1815. He was the discoverer of uranium, and he confirmed and completed the discovery of tellurium and titanium. He likewise discovered zirconia and mellitic acid.

---

*Observations on an extraordinary Chronic Hydrocephalus;* by Dr. Montain, of Lyons.—The chronic hydrocephalus consists in the accumulation of a greater or less quantity of water in the interior of the cranium, as the etymology of the word from υδωρ and κεφαλη denotes. This disorder must not be confounded with that which has improperly been denominated Acute Hydrocephalus, which is only a phlegmosis of the brain, or its membranes, accompanied generally by a greater exhalation of serosity.

The hydrocephalus which really merits the name, accompanies the infant into the world, or developes itself in the first stages of infancy, the disorder is always mortal, and rarely permits the infant to survive the first months or years of existence; the cranium acquires by degrees a considerable size; the face, which does not partake of this development, assumes a singular character, and often imposes on credulous minds, who fancy they discover in this singular conformation traces of the resemblance of different animals,—whence a new source of the belief in monstrosities.

The hydrocephalus which forms the subject of the present observation, is of a young girl, ætat. 19. She was born in the hospital of the Charity at Lyons: it appears that she brought into the world the seeds of this disorder, which progressively developed itself to the present time, when it has acquired an extraordinary magnitude. The following is a faithful physical and moral portrait of this girl.

She is rather below the middle size; her eyes are animated and well placed, but rather too far distant; she is fresh coloured; her features are regular, and her physiognomy does not present the singular and repulsive aspect of others afflicted with this disorder, it harmonizes with the development of the cranium; her head shakes as in old age, which is owing to its weight; the dimensions of the head are as follow:—

The circumference in the line which passes by the eye-brows, the temples, and the occiput, is two feet eight inches; the demi-circumference from the lower part of the occiput to the nose, is one one foot six inches; the demi-circumference from the apophysis mastoides to the opposite one, nearly two feet.

The cranium is solid, and offers no marks of fontenelle or suture, as is usually discovered in such patients.

Her mind and disposition do not appear to be altered by this infirmity; she is pretty intelligent, has a good memory, her judgment correct, and her replies even witty. Her mind has not been much cultivated, the mode of instruction hitherto adopted towards orphans being very confined, but she reads tolerably well.

I insist the more on this degree of intelligence and instruction, as I know no other example of a person afflicted with this disorder of her age, who presents such a phenomenon, as the greater part of children so afflicted are in a greater or less state of idiotcy.

The health of this girl is at present good; she is often afflicted with cephalalgia; her menses are perfectly established. I have attended her since the age of fourteen. She has successively had the itch, an inflammatory and an adynamic fever, which have all terminated happily.

M. Roux, surgeon-in-chief, adjunct at La Charité of Paris, and M. Delpech, professor at Montpellier, have examined, with great care and curiosity, this phenomena, in their passage through Lyons.

CLAUDIUS MONTAIN.

---

*Employment of the Extract of Nux Vomica in Cases of Paralysis.*—At each sitting of the Faculty of Medicine at Paris, new facts are stated as to the effects of this popular medicine in cases of paralysis. In the sittings of the 27th of February, Dr. *** related what was conceived an astonishing circumstance, that he had administered to two women in his hospital as much as fifty grains each per diem. At the sittings of the 6th instant (March), he publicly begged to correct his assertion, as the women, he said, had deceived him; they confessed, that they were not such fools as to swallow all the things the doctor ordered, and, as soon as the religieuse who administered the pills had turned her back, they were in the habit of spitting them out; so that, the doctor said, instead of fifty grains per diem, he could not say, with any degree of certitude, that the women had taken more than from twenty to twenty-two grains each per diem. He caused the extract to be administered in a lavement, which had, as usual, produced beneficial effects in restoring to paralysed members their natural action; but, it must be carefully inculcated, that, in the administration of this powerful medicine, it must be suspended the instant it produces spasmodic affections.

---

*Singular Example of Lameness cured by a Fracture.*—A Roman was threatened with suffocation from matter formed in the breast. In despair, he sought death in battle; the wound of a lance opened the abscess, and the wound, which was thought mortal, saved his life. An accident produced a cervical luxation in a person, and his head was constantly turned on one side; another accident cident turning it violently the other way re-established it in its natural position. C*** had his leg fractured; he was attended by N***; the accident left a frightful deformity; the bones were already united, when, having a frightful dream, he leaped out of bed and broke the leg anew in the same place; he took care not to send for N***; another surgeon reduced the fracture properly, and left no trace of the deformity. These are circumstances in which accidents have been favourable; doubtless, many more might be quoted. The following will show how they may, in certain cases, be profited by.

A man of the name of Bernard had his thigh broken at the age of twenty; it was shortened nearly three inches, and he halted considerably. At the age of sixty, he had the other thigh fractured, it was reduced, and the thigh re-established in its natural length. A fortnight afterwards, he was brought to my hospital. At first, I thought it was the other thigh that was fractured, on account of its being so much shorter; having examined the new fracture, I resolved on separating the fragments anew, and I shortened this thigh three inches. The man was perfectly cured, and his inferior members were become equal, so that the man, until twenty, walked straight, and was five feet six inches high until the age of twenty; he halted for the next forty years; and, in the decine of life, he walked perfectly straight, with the loss only of three inches of his height. CL. MONTAIN,

Chirurgien en Chef de l'Hospice de la Charité de Lyon.

---

*Sea Water rendered Potable.*—This grand desideratum seems at length to be, in a great measure, attained by simple distillation. The French chemists have been unable to discover, in distilled sea water, any particle of salt or soda in any form; and, it is ascertained, that one cask of coals will serve to distil six casks of water. This is highly important. A vessel going on a voyage of discovery by order of the French government, commanded by the celebrated navigator, M. Freycinet, will only take fresh water for the first fortnight; but, instead thereof, coals, which will be only one-sixth of the tonnage; the distilled sea water being found perfectly as good as fresh water that has been a fortnight on board.

---

*Note on a General Transposition of the Viscera, observed by* M. BECLARD.—A case of general transposition of the thoracic and abdominal viscera has been found at the laboratories of the faculty of medicine, in the body of a woman of about fifty, who died of a pulmonary affection. In this subject, the point of the heart corresponded with the interval of the sixth and seventh true ribs on the right side, the liver was lodged in the left hypochondria; the spleen in the right ditto; the stomach had its pyloric orifice pointed to the left, and its large extremity to the right. In short, there existed a general transposition of the viscera from right to left, and *vice versâ.*

M. Sabatier, in a memoir read to the Academy of Sciences, has remarked, that, in nearly all individuals, the vertebral column presents sents in the dorsal portion a lateral inflexion or curvature, of which the concavity is to the left, and the convexity of course to the right. This illustrious anatomist had also remarked, that the greater part of those who are hunchbacked, have it to the right. He thought he had discovered, that these two effects depended on the presence of the crosse of the arterial aorta on the left upper part of the dorsal column; he thought that this vessel, by its continual beating, determined the displacing of the vertebræ.

Some anatomists, and, in particular, Bichat, doubted the correctness of this explanation, they thought that the curvature in question of the spine, depended rather on the more frequent use that we are in the habit of making of the right arm; they asserted even, that, in left-handed subjects, this curvature was reversed. A general transposition of the viscera was very proper to terminate this discussion, for, the *crosse* being found on the right of the vertebral column, it is evident that, if the curvature depended on its presence, it ought to be the reverse of what it ordinarily is.

Exactly the opposite of this is the case. M. Beclard, who has had several occasions of witnessing similar transpositions, as well in corpses as in living bodies, has always remarked, that the curvature of the spine remained the same, if the individual availed himself generally of the use of the right arm.

In the present case, this disposition is proved anew; the right arm was stronger and more muscular than the left, consequently there is every reason to believe, that the woman was right-handed. In her the vertebral column was curved in the ordinary way.

M. Beclard, having compared the cases of general transposition with the dispositions presented by deformed hunchbacked or lame persons, has deduced from his observations the following consequences:—

First, There are primitive malconformations. Secondly, The lateral transposition is perfectly compatible with a state of health. Thirdly, Account must be taken of this transposition in the diagnostics of acute disorders. Fourthly, That it exists probably in the ratio of 1 to 6000. Fifthly, That the ordinary predominancy of the action, and the nutrition of the right arm, does not depend on its receiving the blood more directly from the heart than the left arm. Sixthly, That the lateral curvature of the vertebral column does not depend on the presence of the *crosse* [bend] of the aorta, as M. Sabatier believed, but on the predominance of action and nutrition of the right arm. Seventhly, That the frequent curvature to the right in hunchbacks, and the accidental elevation of one shoulder, depend on the same cause, or on the irregularity of the length of the inferior members.

We may add to these judicious reflections, that not only it is not necessary to force children to use their right hand in preference to the left; but more, that it is dangerous to do it, because it may contribute to destroy the rectitude of the vertebral column; and, that it is very important to forbid the use of the right hand to children whose spine begins to deviate from its proper form.

 *** We

*** We have already given a brief notice of the above transposition from the notes of our correspondent, when the subject was exhibited, the physiological and pathological observations contained in the preceding note have induced us to give it to our readers. [The last-mentioned paper has not reached us.]

---

On Tuesday, the 11th, Dr. SPURZHEIM gave his introductory Lecture. It was painful to hear him introduce the subject of the treatment he has received from some of our Reviewers in Great Britain. It, however, served as an opportunity of illustrating his design, by contrasting it with the opinions they have foisted upon him. He began by admitting that we know nothing of mind but by its effects, any more than of matter but by its properties. That, though he undertook to shew, by certain marks, the dispositions of certain individuals, yet, this by no means proved what their actions would be. That most men have certain propensities, no one can question, or that these propensities may be resisted. If we may believe the history of Socrates, certain propensities were manifested in the countenance, yet in him they were resisted by philosophy. That they may be resisted by religion, we have daily proofs, from the altered characters of certain individuals. It should be recollected, too, that, in the same individual, we find dispositions, as we should previously conceive, totally inconsistent with each other. This has been beautifully attended to by our best poets, and is universally remarked, by those who have studied the human character, and what most men will discover, if they attentively examine themselves. This can only arise from a variety of propensities; and whether these are manifested by any particular marks, remains to be proved. That nothing of the kind has been hitherto discovered may be easily accounted for, because the inquiry has not hitherto been undertaken. We have, as Dr. Spurzheim observed, been more attentive to other animals than to man. Even in our examinations of these, we have lately been more attentive to classification than to the minute examination of their habits, and to any peculiarities of structure with which those habits are attended, in different species of the same genera.

In proof of the greater attention to the brain which Gall and the lecturer had paid, he produced the figure of a hydrocephalic subject, whose head measured thirty-three inches. Yet this subject retained her senses, her affections, and a degree of rationality. All this is now explained, by showing that the brain is not absorbed, as is usually supposed; but that its fibres are extended horizontally, instead of shooting in the perpendicular or vertical direction. Some casts of the craniums of idiots were produced, which were particularly small, and in which the surface was so smooth as to show nothing of that character which may be distinguished in the skulls of other men.

To show how accurately these subjects were marked by the ancient sculptors, a cast of the anterior part of the head of Socrates, and another of Jupiter, was produced, from poetical fiction, or philosophical philosophical observation. The forehead of the *pater hominumque Deumque* was peculiarly spacious.

The cerebellum, which these philosophers consider the organ of physical love, he shows, in all animals, is largest in the male. In the human there is some variety, but the general analogy is the same. The organ which marks the love of offspring; on the contrary, is largest in the female, excepting in those animals who take a joint interest in the care of their offspring. In these it is similar. In the human, the variations are similar to those of the former manifestation. He dwelt, with much propriety, of the difference between those organs; the manifestations were well ascertained, and such as required further experience. His class is respectable, especially when we consider that his intentions were not generally known. The attention paid him was still more honourable, so that we have great reason to hope the London as well as the Edinburgh scholars will maintain their credit, in their treatment of a foreigner whom their writers have endeavoured to stifle as soon as he appeared.

The demonstrations of the recent brain were made at the Theatre in St. Bartholomew's. Dr. S. having fortunately procured the contents of two different crania, was enabled to show the difference of various parts, and the comparative magnitudes of each. In one the cerebellum was largest, and in that the pons also. In the other the lobes were largest. The brown matter in the pons varolii and its fibrous structure, with the dissection of the fibres, in the manner pointed out by Gall and himself, were satisfactorily demonstrated. The *crura* of the brain were exhibited as they enter the lobes, with their gradual increase, and the part at which they assume brown matter, with the gradual increase of each, and their progressing fibrous form, to the extremities of the convolutions. The progress of the optic nerve was traced, and shown to be different from the common opinion as to the thalami. The connexion of the parts of the brain was exhibited, and an explanation of the manner in which the various organs are detected on the surface, which is somewhat similar to the expansion of the optic nerve on the retina, the auditory on the bones of the ear, the gustatory on the mouth, and the olfactory on the nose. By diverging and converging fibres, Dr. S. understands, 1st, such fibres as communicate with the medulla oblongata; and 2dly, such as unite the various parts of the brain. The delicacy of the examination, and the novelty of the subject, added to the number of his hearers, to all of whom he thought it right to give an accurate demonstration, prevented his completing the subject in the two demonstrations he has given. We shall attempt to follow him in our next.

Hitherto Dr. Spurzheim has maintained his ground in the examination of the brain so much to the satisfaction of his hearers, that we trust, in London, as in Edinburgh, the character of the nation will be maintained by the public voice, if sullied by its popular writers.

On

On the 8th of March, the Medical Society of London celebrated their 44th anniversary, which was very respectably attended. The scrutators reported that the election for officers and council had fallen upon the following gentlemen, viz.

*President*—Dr. Walshman.

*Vice Presidents*—Dr. Temple, Dr. Merriman, John Abernethy, Esq. George Young, Esq.

*Treasurer*—John Andree, Esq.

*Librarian*—Dr. Hancock.

*Secretary*—Henry Blegborough, Esq.

*Registrar*—Thomas J. Pettigrew, Esq.

*Orator for* 1818—Dr. Uwins.

*Council*—Drs. Adams, Babington, Clutterbuck, Hamilton, Laird, Pinckard, Uwins; Messrs. Norris, E. Leese, B. Atkinson, T. J. Pettigrew, Mathias, J. Taunton, Damant, Bartlett, Johnston, Sutliffe, Stevenson, E. Austin, Edwards, Hooper, Beveridge, Wachsel, Heath, Vauce, Whateley, R. Adams, R. James, H. C. Field.

The report of the finances, and improvement in the library, was very favorable. The second part of the first volume of the Transactions of the Society was announced as ready to be delivered to the Fellows.

---

A curious optical phenomenon has appeared at Liverpool. As the subject is under strict inquiry, we forbear to say more till properly authorised.

---

Dr. Adams is informed that there is some error in the account of the establishment of the Veterinary College, as stated in his Life of Hunter. He will be thankful for any information on the subject which will enable him to correct such error.

---

Dr. Merriman and Dr. Ley will re-commence their Lectures on Midwifery, and the Diseases of Women and Children, at the Middlesex Hospital, on Monday, April 21st, at half-past ten o'clock.

---

Mr. Curtis, Aurist to his Royal Highness the Prince Regent, and Surgeon to the Royal Dispensary for the Diseases of the Ear, will commence his next Course of Lectures on the Anatomy, Physiology, and Pathology of the Ear, on Thursday, the 17th of April, at seven o'clock in the evening, at his house, No. 18, Soho-square.

---

Sir William Adams is about to publish, A practical Enquiry into the Causes of the frequent Failure of the Operations of Extracting and Depressing the Cataract, and the Description of a new and improved Series of Operations, by the practice of which most of these causes of failure may be avoided.

---

A new edition of Dr. Thomson's System of Chemistry is in the press, and will speedily be published. The work will be entirely re-modelled, and will be comprised in four octavo volumes.

Mr. JOHN BELL has a new work in the press, in one vol. royal octavo, entitled, the Consulting Surgeon, which is nearly ready for publication.

---

Speedily will be published, A Medico-Chirurgical and Biographical Chart of Medical Science, from Hippocrates to the present time, exhibiting, in a condensed form, the progress and present state of that science; with short Biographical Notices of the most eminent Authors, in this and other countries, who have contributed to enhance our stock of Medical Knowledge.

---

In a few days will be published, the Cottager's Companion, intended to instruct the labouring Poor in the Art of Cottage Gardening; to which is added, a Description of upwards of 50 Wild Plants that are useful for Food, being one of the objects of the Œconomic Institution; by W. SALISBURY, Author of the Botanist's Companion, Hints to the Proprietors of Orchards, &c.

---

### REPORT OF DISEASES.

NOTHING particular has occurred during the current month, but a continuance of catarrh and other inflammatory diseases. The spring, though sickly, has been much less so than usual. Even the sudden setting-in of cold frosty weather, after a mild winter, has produced effects as temporary as the change itself.

Children, however, have suffered very seriously. Acute hydrocephalus, (as it is called,) or inflammation of the brain, has been very frequent and very fatal. It is difficult to ascertain why the same remedies should not always be successful; and still more difficult to determine whether a physician's directions are always followed with the same attention. But, in cases where the Reporter has had every reason to believe his directions have been attended to, and where he has had certain proofs that the expected evacuations have followed the application of his remedies, he has found all his endeavours ineffectual; whilst in most others, apparently similar, a similar treatment has been followed with the most flattering success. The plan has been cupping, leaching, and strong doses of the submuriate of mercury. Two or three interesting cases have occurred in the latter part of the month, the issue of which will be given in our next Number.

A remarkable case of plethora occurred in a young adult. He was for some days threatened with apoplexy, and cupped. The symptoms returned, when, on a sudden, he was seized with the most violent symptoms of Tracheitis, during which he was unable to articulate, though capable of expressing his wishes in writing. By copious bleeding, instantly used, and other evacuations, he recovered.

